# The Ultrastructural Effects of Sulfachloropyrazine on *Toxoplasma gondii* Tachyzoites

**Published:** 2013

**Authors:** YB Zeng, H Dong, HY Han, LL Jiang, QP Zhao, SH Zhu, WJ Ma, J Cheng, B Huang

**Affiliations:** Key Laboratory for Animal Parasitology of Ministry of Agriculture, Shanghai Veterinary Research Institute, Chinese Academic of Agricultural Science, Shanghai 200241, China

**Keywords:** Sulfachloropyrazine, *Toxoplasma gondii*, Tachyzoites, Ultrastructure

## Abstract

**Background:**

Toxoplasmosis is one of the most common parasitic infections of humans and other mammals. This study was aimed to understand the mechanism of action of veterinary medicine-sulfachloropyrazine (SPZ, 99.97%) against *Toxoplasma gondii*.

**Methods:**

*T. gondii* tachyzoites were soaked in PBS (as a control) or SPZ (250 mg/mL) for 2 h at 37 °C. After being processed, any ultrastructural changes of the tachyzoites that had occurred were observed by Scanning Electron Microscopy (SEM) and Transmission Electron Microscopy (TEM).

**Results:**

The tachyzoites from control groups with a uniform size had a smooth surface and intact cell or nuclear membranes. In addition, an oval-shaped nucleus, conoids and micronemes were also observed. By contrast, many parasites from the SPZ-treated groups were detrimentally affected by the treatment. Some appeared to be of the vacuolization in their cytoplasm, with the substantial reduction in the number of dense granules and the blur of some organelles.

**Conclusion:**

The morphology and ultrastructure of tachyzoites can be affected significantly by SPZ, which might kill the parasite by inhibiting its energy metabolism, inducing apoptosis and damaging its structure****. The study provides an experimental basis for further study on the mechanism of SPZ against *T. gondii*.

## Introduction

*Toxoplasma gondii* can cause important parasitic infections of man and other mammals. It can result in major public health problems, with a high socioeconomic impact in terms of human suffering, including the cost of caring for sick, developmentally delayed and blind children (1). On farms, *T. gondii* can cause severe congenital diseases and abortions in farm livestock (2); therefore, it is also a significant cause of reduced profits in livestock agriculture (3–5).

At present, there were no effective drugs to prevent clinical toxoplasmosis in animals (6, 7). Sulfachloropyrazine (SPZ), a sulfonamide, has been reported to be effective in preventing and treating coccidiosis in chickens, and widely used in poultry industry since 1970s for its low cost and relatively high efficiency (8–12). In previous studies, SPZ was found to be a therapeutic option for *T. gondii* infection in an acute murine infection model. Oral application of SPZ (twice daily for a period of 5 days) in *T. gondii*-infected mice was effective in achieving significant reduction of the parasite burden (13, 14).

The present study was carried out to observe the cytologically visible effects of SPZ *in vitro* on tachyzoites of the RH strain of *T. gondii*.

## Materials and Methods

### Animals and Parasites

Outbred, female strain KM mice were used in each experiment according to the Animal Welfare Regulations of World Organisation for Animal Health (OIE) and European Union (EU). The RH strain of *T. gondii* was propagated intraperitoneally at 3-or 4-day intervals in the mice. The parasite-rich peritoneal fluid from infected mice was washed in phosphate-buffered saline (PBS) three times and passed through a 27-gauge needle with a 5.0 µm-pore filter (Millipore, Bedford, MA). Pure tachyzoites were counted using a hemacytometer and diluted to 1×10^8^/mL (PBS, pH7.2) for further use.

### Drugs

Sulfachloropyrazine-sodium (SPZ, 99.97%) was produced by Shanghai Baoshan Zhenzong Biochemistry Engineering Factory, Shanghai, China. The agent was provided in the powder form of the pharmaceutical raw material.

### Scanning and Transmission Electron Microscopy Process

Drug trials were carried out in eight-well plates, with each well containing 1×10^8^ organisms. Half of the plates contained SPZ (at a concentration of 250 mg/mL, dissolved in PBS) and the other half contained only PBS. The parasites were incubated at 37 °C in a 5% CO_2_ atmosphere for 2 h. After incubation, the samples were washed twice in PBS and centrifuged at 1000 rev/min for 10 min at 4 °C. The sediment was pre-fixed with 2.5% glutaraldehyde for 8 h at 4 °C, rinsed three times with 0.1 M PBS (pH 7.2), post-fixed with 1.0% osmium tetroxide (OsO_4_) for 2 h, and washed three times with 0.1 M PBS (pH 7.2). The tachyzoites were dehydrated by sequential incubations in increasing concentrations of ethanol (one time in 30%, 50%, 70%, 90% and 95%, and three times in100%) for 10 min.

For Scanning electron microscopy (SEM) analysis, the dehydrated parasites were soaked with isoamyl acetate for 1 h at room temperature, air dried under a fume hood, sputter-coated with gold in a sputtering system, and observed on a JEOL-6380LV SEM. For transmission electron microscopy (TEM) analysis, the dehydrated parasites were soaked in anhydrous acetone for 10 min and then dried after repeating three times. Parasites were subsequently embedded in epoxy resin at 60 °C for 12 h. Ultrathin sections were then prepared with a LKB-V-ultramicrotome; they were stained with uranyl acetate and lead citrate and viewed with a JEM-2100 TEM (15, 16).

## Results

**Fig. 1:**
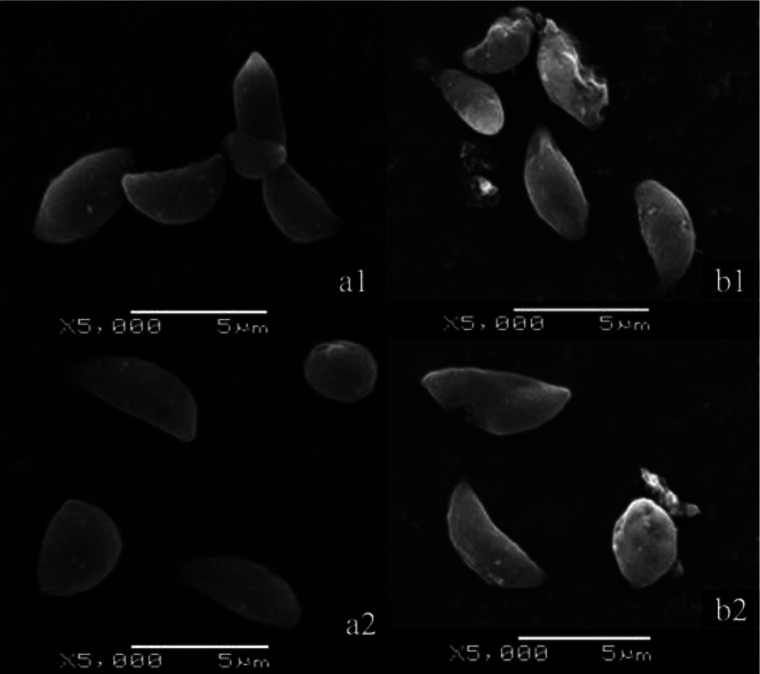
Morphological changes of SPZ on *T. gondii* tachyzoites as visualized by SEM. PBS-cultured parasites (a1 and a2) versus tachyzoites treated for 2 h with an SPZ solution (b1 and b2)

SEM images showed that the tachyzoites in control groups were bow-shaped and of normal size (3-4 µm), the posterior end of each tachyzoite was a smooth curve (Fig. 1-a1 and 1-a2). However, after 2 h of SPZ treatment, the graphs showed that many parasites had an uneven surface with wrinkles and creases, and an incomplete membrane. The different levels of light reflection could be observed among the parasites. In some cases, the SPZ had caused them to disintegrate. (Fig. 1-b1 and 1-b2).

**Fig. 2:**
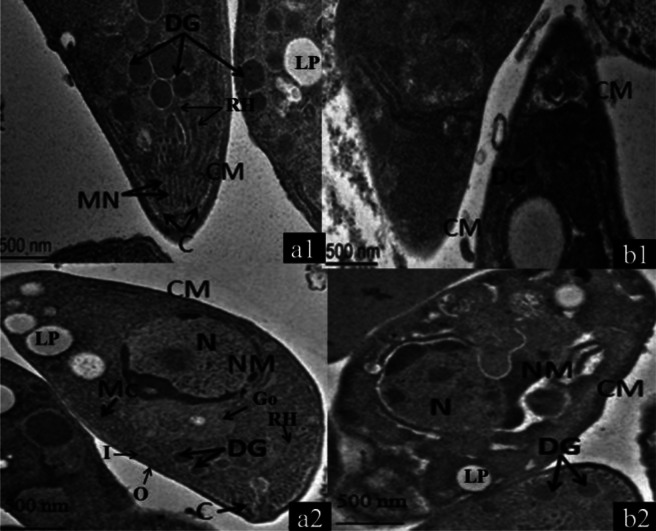
Ultrastructural effects of SPZ on *T. gondii* tachyzoites as visualized by TEM. TEM pictures were prepared with untreated tachyzoites from the PBS-cultured groups (a1 and a2) and with tachyzoites treated for 2 h with an SPZ solution (b1 and b2). Abbreviations: C, conoid; CM, cell membrane; DG, dense granule; Go, golgi complex; LP, lipid body; Mc, mitochondrion; MN, microneme; N, nucleus; NM, nuclear membrane; RH, rhoptry; I, inner layer; O, outer layer

TEM revealed that PBS-cultured parasites retained the normal morphological features of tachyzoites, such as cell membrane, conoids, dense granules, micronemes, mitochondria, nucleus, rhoptry, lipid body, golgi complex and so on (Fig. 2-a1 and 2-a2). They also had a pointed head and coarse tail according to the normal features. The double membranes of both the nucleus and cells were intact. Ellipse-shaped nuclear and normal chromatin could also be observed. In addition, the mitochondria and numerous dense granules in the body could clearly be seen. The conoids and micronemes were distributed in an orderly manner at the head of the parasite. By contrast, after 2 h of SPZ treatment, the parasites had sustained serious damage, including condensed chromatin being compressed against the nuclear envelope, and cytoplasm association with abundant vacuolization. In addition, the number of dense granules had decreased and the conoids had become blurred. Some tachyzoites had lost their structures completely, including dense granules, mitochondria, cell membranes, micronemes and nuclear membranes (Fig. 2-b1 and 2-b2).

## Discussion

Electron microscope has been widely used in the classification and identification of parasites, and in exploring the effects and mechanisms of drug action by observing the effects of the drug on parasite ultrastructure. Dubremetz et al. (2009) used ultrastructural techniques to classify previously unrelated parasites within a single phylum, the Apicomplexa (17). Stettler et al. (2003) researched the in vitro parasiticidal effect of nitazoxanide (NTZ) on *Echinococcus multilocularis* metacestodes using electron microscopy (16). Zhou et al. (2010) investigated the effect of diclazuril on the morphology of second-generation merozoites of *Eimeria tenella* using TEM (18).

SPZ is a veterinary medicine available in a numbers of countries worldwide and is widely used for treating poultry coccidiosis (9, 10, 12). However, its activity against *T. gondii* has not been reported. In our previous studies, the efficacy of SPZ against *T. gondii* was confirmed for the first time. Oral application of SPZ in *T. gondii*-infected mice was effective in reducing the parasite burden and prolonging the average survival time and the time-point of 50% fatalities of the infected mice (14, 19).

In the current study, the ultrastructural changes of *T. gondii* treated with SPZ were observed using an electron microscope. The morphology, membrane systems, organelles, chromatin structure and other structures were changed significantly after treatment with SPZ compared with control groups. In all individuals treated with SPZ, characteristic morphological changes that were associated with cells undergoing apoptosis, including condensed nuclear chromatin, cytoplasmic shrinkage, formation of apoptotic bodies with an apoptotic phenotype and loss of mitochondrial function, were observed. Meanwhile, separation of the laminated and germinal layers, cytoplasmic vacuolization and loss of organelles were also observed. We hypothesize that SPZ maybe kill *T. gondii* by inhibiting its energy metabolism, but further study are needed to verify this hypothesis.

## Conclusion

Our results indicate that SPZ effectively inhibits the growth and development of *T. gondii* by changing their ultrastructures, such as nucleus, membrane, conoid, dense granule, and micronemes. This research would provide an experimental basis for further interpreting the mechanism of SPZ against *T. gondii*.
